# Influence and Action Mechanisms of Governmental Relations Embeddedness for Fostering Green Production Demonstration Household: Evidence from Shaanxi, Sichuan, and Anhui Province, China

**DOI:** 10.3390/ijerph191911923

**Published:** 2022-09-21

**Authors:** Lipeng Li, Apurbo Sarkar, Xi Zhou, Xiuling Ding, Hua Li

**Affiliations:** 1 School of Economics and Management, Ningxia University, Yinchuan 750021, China; 2 School of Economics and Management, Northwest A&F University, Yangling, Xianyang 712100, China; 3 School of Food Science and Agriculture, The University of Queensland, St. Lucia, QLD 4072, Australia

**Keywords:** government relationship, embedded, green production, demonstration zone, household cultivation, action mechanism, innovation, diffusion of innovation

## Abstract

As an innovative tactic, the core aspects of green products should be comprehensively demonstrated and firmly promoted to enhance their adoption. For doing so, continuous governmental support and interventions through distinct sets of networking and relationships could be crucial for synthesizing and diffusing the extent of green production demonstration households. Interestingly, the structural relationship between these two has not yet been evaluated comprehensively by the existing literature. Therefore, the study empirically analyzes the impact and mechanism of government relationships embedded in fostering green production demonstration households. The study compiles the empirical data from 963 farmers which were collected from the major tea-producing areas of Shaanxi, Sichuan, and Anhui provinces, China. In order to craft the findings, first we constructed the ordered Probit for benchmark regression analysis. Meanwhile, the Ordinary Standard Error Ordered Probit model, Ordered Logit model, and multivariate linear model were constructed for the robustness test. Third, the Extended Ordered Probit model and Bootstrap mediation effect model were used to test the path diagram. Finally, robustness testing and endogeneity processing test were used to explore the reliability of the findings. The results showed that: (i) Government relationship embedding has a positive effect on fostering green production demonstration households. In particular, factors such as relationships with general government staff, professional and technical personnel, and village cadre are most significant. (ii) Seemingly, the heterogeneity analysis shows that the farmers with large operating scales and low family economic status have a relatively stronger impact. (iii) Further mechanism research results show that government relations are embedded through government identification (policy identification, government trust), improving farmers’ behavioral ability (production knowledge reserve, self-efficacy), and strengthening farmers’ perceived value of green production (self-interest perception, altruistic values). Therefore, the government should strengthen the interactive mechanism embedded with farm households and extend support for green production demonstration zones. The farmers’ information-sharing facilities and platforms should be modernized and highlighted according to the local conditions and long-term targeted strategies.

## 1. Introduction

Since the reforming and opening up in 1978, the agriculture sector of China has witnessed an outstanding and quick transformation phase from traditional to relatively modern agriculture tactics through the greater interaction of technological and social transitions [1,2]. Moreover, several global issues and phenomena, such as climate change, global warming, and frequent weather changes, are largely influencing the availability of environmental friendliness within the core agriculture practices [3]. The agriculture sector is one of the prime releasers of greenhouse gas (GHG) globally, being approximately solely responsible for 27% of the total output [4]. However, with China’s carbon neutrality target for 2060, a transition is in process, especially in a net-zero transition in food and agriculture that would impact how we farm, what we eat, and how we manage our forests and natural carbon sinks [4,5]. Likewise, exploring the possible methods and techniques for the reduction in pollution from agricultural activities has become one of the most discussed topics among industries, academia, government, and international organizations [1,6].

As with most other agricultural products, tea production and distribution are also contributing to massive environmental issues from altering biodiversity, degrading soil, and water pollution to shrinking the forest area which results in a massive impact on climate change and possesses greater vulnerability to its impact as well [7]. Moreover, as the most popular drink after water, the long-term success of tea production relies on consumers and their continued appreciation for the product [8]. In the modern era, consumers are more conscious of how any product is produced and distributed [9]. Particularly in terms of tea production, the concept of green production is delivered by the two basic components of producing tea leaves without harming the environment or being responsible for a lower impact on the environment. Therefore, the notion of green production has emerged which is proven to reduce the widespread depredations caused by agricultural activities [10,11]. Accelerating the promotion of green production techniques is not only a scientific method to effectively prevent and control agricultural non-point source pollution [12], but also it can help to cope with resource depletion and mitigate environmental pressure [13,14]. Seemingly, it also can act as a feasible path to achieving the coordinated revitalization of the rural economy and ecology [15]. Therefore, aligned with the notion of governmental environmental policy, the aspects of green production tactics are currently becoming popular among tea producers worldwide.

However, tea is produced globally by following conventional methods and thus it is challenging to foster green production within this dynamic industry [16,17]. Likewise, the central tea-producing area in China is mostly based in the rural mountain areas where technological transitions mostly seem low [7]. In this situation, the aspects of green production demonstration households can act as a major transitional channel to formulate, foster, and comprehend the core principles of green production tactics [18,19]. In the agriculture sector, the concept of production demonstration households is widely used by developing and developed countries as it helps to showcase any novel form of technology or tools, products, and tactics that could be crucial for any potential adopters [20]. In rural areas, this practice may have greater influence as it exercises and promotes the aspects of “learning by doing” and “learning by watching” tactics [20]. Seemingly, the application of green production demonstration households can dispel the concerns of conventional farmers, and through the “acquaintances” method, it can quickly spread within rural areas and help in the diffusion and application of green production behavior [21,22].

Therefore, how to form green production demonstration households has become a key issue for the government in promoting a green production promotion system and enhancing the adoption level of green production applications. As dynamic and multidimensional aspects, the effective embedding of government relations in the cultivation of green production demonstration households has become a focal point for the government [23]. Moreover, in a society such as China, where mutual understanding, social bonding, and trust in governmental bodies are relatively strong, the notions of innovation are largely fostered by the direct and indirect supervision of governmental bodies. In this context, it is necessary to critically explore the impact, and action mechanism, of the government’s relational embeddedness in fostering green production demonstration households. Relational embedding is the interpersonal interaction of information and favors, which is mostly characterized by trust and cooperation among the actors (subject and objects) in a particular relationship network [24]. In the agriculture sector, it is widely practiced on the supply chain side [25], where the subject of government relationship embedding is the governmental bodies, and the object is the farmers. In the relationship, the government and farmers directly share information and provide support and favors in terms of mutual corporations [26]. According to Van Loon et al. [27], compared with a single subsidy or regulatory measure, government relationship embeddedness has a more leading role and long-term impact on fostering any particular actions. Moreover, it can strengthen farmers’ trust in government and policy recognition, improve farmers’ technical literacy and self-efficacy, promote farmers’ recognition, and eventually enrich their self-interest and self-efficacy [28]. In particular, in a society such as China, it can substantially foster altruism and other values and rectifies the support channel sustainably [1,29].

Interestingly, the existing literature has not paid much attention to critically exploring the interrelationships between the effects of government support and relationship embeddedness in formulating and popularizing green production demonstration households. Which eventually results in the frequent occurrence of “demonstration breaks” and the gradual loss of the diffusion effect of green production within the rural regions [10,30]. In recent years, the existing studies (such as Carter et al. [31], Varela-Candamio et al. [32], and Yazdanpanah et al. [33]) have mainly focused on the direct impact of government subsidies and punishment measures and other resources through the willingness and behavior of general farmers to move to green production. In a study of 800 tea farmers located in the Qinba and Huangshan Mountain regions of China, Yu et al. [34] found that governmental and communal support has certain mediatory effects on fostering innovations and eventually triggers farmers’ proactive behaviors. Akram et al. [35] explored the rural farmers in Punjab, Pakistan, and found governmental relationships have impacted significantly to avail sustainable land tenure and help maintain a smooth transition of governmental policies and support. However, to the best of our knowledge, there are very limited studies that can be found triggering enough attention and effectively explaining the impact of government relations on the formulation of green production demonstration households and green production behavior.

The above-mentioned discussion has led us to the following research questions that need to be explored more critically and comprehensively: (i) what are the key channels by which government can foster relational embeddedness? (ii) What factors may impact the relationships most? (iii) Whether, and how do, the behavioral capabilities and socioeconomic factors and perceptions possess an impact on the relationship between government and the green production demonstration households? Therefore, to answer those research questions, the study constructs a theoretical framework for empirically exploring the interrelationship between the relational embeddedness of government and green production demonstration households. In the theoretical framework, we particularly integrated various core channels and major statutory and behavioral factors that may possess influence in rectifying governmental relational embeddedness based on an extensive literature review. The study uses the survey data of 963 farmers collected from the major tea-producing areas of Shaanxi, Sichuan, and Anhui provinces, China to craft its findings. The principal contribution of the study is to expand the “diffusion of innovation theory” proposed by Rogers [36]. Moreover, the study explores the impact of governmental relational embeddedness and green production demonstration households in an integrated framework. We also explore the subjective aspects (governmental channel, government trust, and policy identification) and objective aspects (farmer’s interpersonal, social and behavioral factors) in a structural framework. It may help policymakers to realize the importance of governmental relational embeddedness in the formation and diffusions of green production demonstration households. By exploring and quantifying the proposed framework, the study is expected to provide several policy suggestions which will lead the government to identify the most important channel. It will support comprehension of how the governmental relationship can be integrated with the rural households to attain central governmental key goals to become more environmentally friendly and maximize the interaction of sustainable development goals.

## 2. Theoretical Framework and Research Assumptions

The prime objective of the study is to explore the connection between governmental relational embeddedness and green production demonstration households in an integrated manner. Governmental relational embeddedness is a form of social embeddedness, that is the connections between socioeconomic activities and interpersonal behavior and includes things such as social communication, having shared common attributes, and ecological protection [37]. According to Xie et al. [38], these types of network embeddedness have significant effects on influencing farmers’ adoption behaviors. The notion of governmental embeddedness has been widely used by researchers working on pollution prevention, resource saving, and farmers’ behavioral studies (such as Mariola [39], Hedberg and Zimmerer [40], and De Lauwere et al. [41]). However, in this study, production demonstration households were explored through green production demonstration households. Green production demonstration households referred to in this study are those households that have a high degree of adoption of green production technology and are quantified by the green production level of the farmers. More specifically, the concept of green production in this study refers to the production of tea leaves without harming or being less harmful to the environment. Its main objective is to reduce pollution, improve and render more effective natural resource utilization to foster reduced waste-water contamination, and eventually aims to protect the ecological environment. The study used the criterion of Li et al. [42] which recommends that the higher the green production level of farmers, the closer they are to the green production demonstration households in the practical sense.

Currently, the tea industries are facing several socioeconomic and environmental challenges, such as the widespread overuse of pesticides and fertilizers [43], the insufficient utilization of agricultural reusable wastes such as tea branches and leaves [16,18], and the difficulty in implementing environmentally friendly technologies such as organic fertilizers and green prevention and control [34]. Therefore, pollution reduction, resource conservation, and environmental friendliness have become important contents of the development of green production behavior among farmers in the tea area, and can also affect the environmental quality, biodiversity, product quality, and many other aspects at the same time.

### 2.1. Government Relations Embedding and Green Production Demonstration Household Cultivation

Green production demonstration households are embedded in the interaction network between farmers and the government [44,45]. This is because government relationship embedding can enhance the interaction between the government and farmers, strengthen farmers’ trust in the government and strengthen the promotion of green production policy recognition, and encourage farmers to adopt green production tactics [46,47]. According to Zhu and Chen [48], government relationship embedding can assist in introducing advanced technical knowledge into agricultural production and systematically improve farmers’ knowledge reserves, effectively alleviating the cognitive constraints, and reducing the obstacles and risk perceptions of farmers. In a review study on government intervention and organic fertilizer promotion, Amgai et al. [49] identified that “supports and interventions from governmental channels and agricultural demonstration zone can successfully transmit the information regarding the policies, incentives and other valuable information which eventually helps in formulating demonstration households”. Sanders [50] explored sustainable agriculture in China and confirmed that the relationship between farmers’ communities and the government fosters a healthy environment which not only helps in extending the capabilities of existing demonstration households but also fosters new demonstration households and eventually triggers the adoption of green production technology. Moreover, several recent studies (such as Xu and Findlay [51], Govere et al. [52], and Buadi et al. [53]) confirmed that the embedded government relationship has the attribute of mobilization, enabling farmers to obtain psychological support and significantly improve their performance. However, self-efficacy motivates farmers to exercise innovativeness, and government relationship embedding can effectively convey self-interested, altruistic, and social values. Interestingly, it has been widely proved that self-motivated and efficient farmers are more likely to adopt innovation and act relatively more responsibly towards the environment [51,54,55]. Given this, the article proposes H1.

**Hypothesis** **1** **(H1).** *The embeddedness of government relations may influence the formulation of green production demonstration households.*

### 2.2. Embedding of Different Types of Government Relations and formulations of Green Production Demonstration Households

According to the core concept of different embedded subjects, government relationship embedding can be subdivided into three forms: relationship embedding with (i) the government’s general staff, (ii) professional and technical personnel, and (iii) village cadre relationship embedding (for more details see Wei and Li [56]). However, government relations can be embedded through various channels, such as government staff, industry professionals and technical personnel, and village cadres which may foster crucial farmers’ production practices [52,57], and can act as support agents in cultivating green production demonstration households [58,59]. In general, governmental relationship embedding can provide farmers with resources and cognitive support, create a viable green production atmosphere, strengthen farmers’ recognition and green production policy awareness, and improve farmers’ green production behaviors’ ability [60,61]. In this process, farmers will be more willing to accept the government’s notion of developing green production demonstration households.

Interestingly, compared with general governmental bodies, professional and technical personnel enjoy higher acceptance among farmers because of their superior technical status and professional knowledge [62,63]. In most cases, they can produce relatively stronger spillover effects and can foster better transitions of innovations. In a study of Taojiang County, China, Qi et al. [64] explored the farmer’s adoption behaviors regarding environmentally friendly fertilizer and found that the relationship between the technical personnel significantly influenced the farmers to adopt certain innovations and led them to spread those among their peers. Adnan et al. [65] investigated Malaysian farmers’ adoption motives for green fertilizer and stated that governmental organizations and technical supports and village cadres all have significant and positive effects on influencing farmers’ adoptions. In a review study of precision fertilizer technology, by highlighting the importance of governmental support organizations, Chen et al. [66] stated that “agricultural extension offices and technical training institutes are tending to work like a knot between the farmers and governments updated policies”. Seemingly, it can be assumed that the information and human relations embedded in their relationships can convey the government’s practical goals and establish the supporting features of government [67,68]. Similarly, the positive image of “service” may significantly enhance farmers’ green production knowledge reserves and the self-efficacy of farmers in implementing green production and strengthened the adoption process [53]. Therefore, compared with general governmental staff, green production demonstration households with embedded professional and technical relationships have stronger perceptions regarding green technology, and demonstration households often foster a certain level of spillover impacts [69,70].

The relationship between village cadres is built based on generational familiarity, close daily interactions, and a high attribute of trust [71,72]. When farmers are practical, they witness the effectiveness of any new technology and it can make farmers agree with the government in understanding the practical value of green production and enhancing farmers’ confidence regarding green production adoption [73,74]. It also implies farmers’ willingness to accept green production policy arrangements [75,76]. In a study of organic fertilizer in Hubei province, China, Yang et al. [77] identified that the village cadres were not only triggering the social acceptance of eco-friendly fertilizers but it has also created learning by creating opportunities for farmers. Similar studies (such as Lu et al. [78], Smol [79], and Avane et al. [80]) have identified the village cadres in fostering effective fertilizers application behavior. In a society such as China, familial ties and kinship traditionally have a significant influence when it comes to adopting any innovations such as new technology or new resources. Though there are many tools for discipline, such as the Party discipline rules, state legislation, and structures for evaluating cadres, it has been hard to hold village cadres accountable [81] in modern China, and especially within the agricultural sector it may rectify the important aspects of transforming the farmer’s behavior [82]. Therefore, it can be assumed that the role of green production demonstration households embedded in the relationship between village cadres is stronger. Based on the above-mentioned discussion, the article proposes H2 as follows.

**Hypothesis** **2** **(H2).** *The relationship between general government staff, professional and technical personnel, and village cadres may impact* *fostering the embeddedness of green production demonstration households.*

### 2.3. Differences in the Role of Government Relations Embedded in the Cultivation of Green Production Demonstration Households under Different Conditions

The existing literature (such as Zhang et al. [83], Yu et al. [84], and Wang et al. [85]) showed that the characteristics of the household head, family management, and family status are the basic environment for the embedding of government relations to foster green production demonstration households, which may cause group differences. Therefore, it is necessary to carry out further analysis to better identify the characteristics of farmers under the embeddedness of government relations that can assist in understanding the formulation and development of green production demonstration households.

Age is a key component of the characteristics of the head of the household [86,87]. In a study of Bangladeshi farmers’ attitudes towards sustainable agricultural practices, Sarkar et al. [88] found that, compared with young people, older farmers are less able to accept and understand innovation, and are also reluctant to participate in government green production training, policy presentations, and other activities. The interaction of information technology and the internet familiarity of household heads is an important component of the information literacy of household heads [89]. Bozorgparvar et al. [90], Wang et al. [91] and Zhao et al. [92] found that the farmers who use the internet generally have a more open attitude towards new things than those who do not use the internet, and have a higher level of awareness of green production and smoother communication with the government. However, existing studies have shown that the role of green production demonstration households embedded in government relations is gradually increasing [93,94]. Given this, we propose H3.

**Hypothesis** **3** **(H3).** *The age of the household head and the knowledge regarding internet usage may impact the formulation of governmental relations embeddedness for fostering green production demonstration households.*

The scale of management and the degree of non-agricultural income are important indicators of the characteristics of family management. Compared with ordinary farmers, farmers with large operating scales are more capable of green production behaviors [83,95], more dependent on the government, and likely to form a two-way reciprocal relationship in the interaction with the government [96,97]. Paudel et al. [98] identified that, as the degree of non-agriculturalization increases, agricultural production is gradually marginalized in household livelihoods. It appears that various studies (George [99], Wang and Liu [100], Jeon [101], and Dorosh and Thurlow [102]) have confirmed that as the degree of non-agriculturalization has improved, so the role of green production demonstration households embedded in government relations has gradually increased. Given this, this paper proposes H4.

**Hypothesis** **4** **(H4).** *Households’ farming scale and income from the non-agricultural source may have an impact on green production demonstration households embedded in government relations.*

Familial technical status and knowledge, economic status, and political status are extremely important to farmers’ behavior [103,104]. In the context of embedded government relations, households with a high-tech status are more aware of the necessity of green production and have the technical ability to take the lead in implementing green production. Farmers with high-economic status have higher incomes and have the economic ability to take the lead in implementing green production [105,106]. Similarly, farmers with high family political connections and status have a higher level of ideological awareness and are more able to bear the responsibility of being in the vanguard of green production [82]. Therefore, as the status of farmers improves, the role of green production demonstration households embedded in government relations gradually increases. Because of this, this paper proposes H5.

**Hypothesis** **5** **(H5).** *The improvement of the technical, economic, and political status of farmer households may impact fostering green production demonstration households.*

### 2.4. The Mechanism of Action Embedded in Government Relations in the Cultivation of Green Production Demonstration Households

The social environment can effectively activate the intrinsic traits of individuals and then affect their behaviors [107]. As an important form of social environment, government relationship embedding can effectively activate the inherent characteristics of farmers and affect whether they can become green production demonstration households [108,109]. This part of the study analyzes the embedded mechanism of government relations in the cultivation of green production demonstration households from three aspects: government identification, behavioral ability, and perceived value.

Government identification is the degree of farmers’ approval of the government itself, or government policies [110]. The embedded government relationship can play a role in cultivating green production demonstration households through enhancing farmers’ government identification by utilizing government trust and policy identification [111]. This is because, first, government relationship embedding can cultivate farmers into green production model households through trust in the government. The embedded government relationship enables the farmers to understand the government intuitively and realize the transformation from unfamiliar to familiar alongside the government [112]. The government can also effectively collect farmers’ demands and formulate response measures in turn, thereby enhancing farmers’ trust in the government, then, increased government trust enhances farmers’ respect and obedience to the government, so that farmers form an optimistic expectation that includes government actions, and encourages farmers to develop into green production demonstration households [113]. Second, policy approval refers to the degree of individual approval of the policy. Whether the policy is approved by farmers depends on whether the policy itself is fair and whether it can be implemented [114]. The poor communication between the government and farmers is an important reason for farmers’ lack of approval or even passive resistance to the policy [27]. The government relationship embedding introduces detailed policy information to improve farmers’ recognition of green production policies [115], so that they form positive expectations for green production [116], and then encourage farmers to develop into green production demonstration households [117]. Given this, this article proposes H6.

**Hypothesis** **6** **(H6).** *Government identification (government trust, policy identification) may have an impact on fostering green production demonstration households.*

Behavioral capability represents the possibility and confidence of farmers to achieve their goals, and government relationship embedding may play a fostering role in cultivating green production demonstration households by improving farmers’ behavioral capabilities, such as by improving their production knowledge reserves and self-efficacy [118]. This is because, first, the lack of production knowledge reserves is an important reason for hindering farmers ‘ green production adoption [29]. Government relations are embedded with highly heterogeneous agricultural production knowledge and concepts [119], which can improve the effectiveness of farmers’ information acquisition, such as field demonstrations. In-depth interactions, such as face-to-face communication, can break the cognition shackles of farmers, and encourage farmers to develop into green production demonstration households [1]. Second, self-efficacy refers to the individual’s belief in whether the self can effectively implement the plan, and individuals with high self-efficacy perform better [88]. The embedding of government relations can provide resources and emotional support and turn this support into the sustainable competitiveness of farmers [120], which can significantly improve farmers’ confidence in dealing with green production risks and self-efficacy in the successful realization of green production [121], and encourage farmers to develop into green production demonstration households. Given this, the article proposes H7.

**Hypothesis** **7** **(H7).** *Farmer’s behavioral capabilities (production knowledge reserves, self-efficacy) may have impacts on formulating green production demonstration households through governmental relationship embeddedness.*

Perceived value is an individual’s overall evaluation of a certain behavior, which includes three dimensions: self-interested value perception, altruistic value perception, and social-interested value perception [50]. The embeddedness of government relations may play a critical role in cultivating green production demonstration households by shaping the perceived values of farmers. This is because, first, government relations are embedded in deeply shaping farmers’ perceived value of green production by creating more value propositions than the conventional methods [122]. The embedding of government relations also may enhance the interaction between the government and farmers, such as policy publicity, training, daily communication, etc. It is more viable when the notion of “Move with emotion, understand with reason” can be integrated with the existing interaction for awakening farmers’ economic, social, and ecological rationality, and ultimately improving farmers’ production knowledge. It is the phenomenon of asymmetry which may significantly shape the perceived value of farmers’ behavior, while government embeddedness usually targets the removal of the barriers by empowering farmers with recognition and enhancing social acceptance [123]. Second, farmers’ green production may need comprehensive self-interest, altruistic value, and social value [124]. When farmers perceive that green production can bring sufficient benefits to themselves, others, and society, they will take the lead in implementing green production [90]. Because of this, this paper proposes H8.

**Hypothesis** **8** **(H8).** *Farmer’s perceived value (perception of self-serving value, perception of altruistic value, perception of social value) may have impacts on fostering green production demonstration households through governmental relationship embeddedness.*

## 3. Methodology

### 3.1. Methods

**Figure 1:**
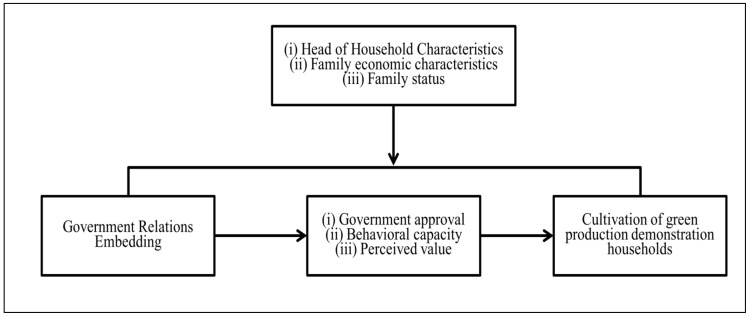
Theoretical framework.

The dependent variable of green production demonstration households changes from low to high, which is an ordered variable, so it is suitable for estimation by the Ordered Probit model, which has been widely used by the related literature (such as Hassen [125], Teklewold et al. [126], and Lanfranchi et al. [127]). It is very popular among agro-economists as it can effectively avoid the estimation error of the multiple linear regression model and improve the estimation accuracy of the model [128,129]. Figure 1 depicts the theoretical framework of the study. The specific models and techniques used in the study are stated below:

First, the theoretical framework of the embedding role of government relations in fostering green production demonstration households was established and tested by the Ordered Probit model, based on benchmark regression. Second, we empirically tested the role of government relationship embeddedness in the cultivation of green production demonstration households and compared them by type. In this stage, the interaction term method and Extended Ordered Probit model were used to test the heterogeneity and reveal the boundary conditions of the embedded role of government relations and render the research conclusions more practical. Third, the interaction term was introduced to construct a moderating effect model to test the heterogeneity of government relationship embedding in the cultivation of green production demonstration households. Fourth, the mediation effect model was used to explore the path through which government relationship embedding plays the role of green production demonstration households, revealing their mechanism of action, and providing a specific reference for government policy formulation and future research directions.

After that, the Bootstrap mediation effect model was used to test the path through which government relationship embedding promotes the cultivation of green production demonstration households. The Bootstrap method has become popular in recent years and it was used to test the mediation effect, which can effectively solve the problem of the “masking effect” reflecting the real impact of the variables, and effectively revealing the mediation mechanism [130,131]. Finally, robustness testing and endogeneity processing were carried out by employing a combination of the Ordinary Standard Error Ordered Probit model, Extended Ordered Probit model, Ordered Logit model, and multivariate linear model which were constructed to confirm the findings of the benchmark model, and so the conclusions drawn in the first part were reliable.

The study used Stata 15.0 (Stata Corp., College Station, TX, USA) to complete the Ordered Probit model, robustness test, endogeneity processing, moderating effect model, and mediation effect model. Stata is an integrated multi-departmental statistical analysis software that is very popular as it has an easy-to-use interface, speedy and reliable output, a total customization option, and comprehensive technical support [132].

### 3.2. Data Sources

To ensure the validity of the data, random interviews were used to determine the survey objects, and the survey objects were limited to farmers over the age of 18 who were familiar with the production and operation of their families, as suggested by Meyer-Aurich [133]. The data were comprised of a survey among farmers in the key tea areas in Shaanxi, Sichuan, and Anhui from July to August 2020. Based on considering the economic development of the county, the level of agricultural development, and the feasibility of investigation, 10 key tea-producing counties (urban areas) were firstly identified, including Xixiang County, Ziyang County, Baihe County, and Hanbin District in Shaanxi; Wangcang County, Tongjiang County, and Emeishan City from Sichuan province; Jinzhai County, Qimen County, and Huangshan District in Anhui. After that from each county (city) we randomly selected 3–4 townships, and from each township randomly select 2–4 villages, each. Finally, from those villages, we randomly selected 10–15 tea growers. Similar to the existing studies (Li et al. [122], Deng et al. [114], and Senger et al. [134]), this study used family characteristics, production situation, cognitive situation, external environment, and green production demonstration as the key indicators. In addition to the household questionnaires, village cadres were also asked to fill out the village-level questionnaires. A total of 1020 questionnaires were distributed to farmers, and 963 farmer households were obtained after excluding the questionnaires due to farmers leaving the area and missing key information. The effective rate of the questionnaires was 94.41%. The distribution and capacity of the sample farmers are shown in Table 1.

### 3.3. Variable Selection

This study adopted the model’s variable according to the core concept of the theory of diffusion of innovations developed by Rogers et al. [135] and adjusted those according to the core setup of the study. In this study, the design of the variable of green production demonstration household was derived according to the role of the adopter and the acceptance of innovation. The specific assignment of the green production demonstration household variable was “Opponent = 1, Non-Implementer = 2, Late Implementer = 3, Early Implementer = 4, Earliest implementer = 5”. Among them, the opponent refers to the peasant household that has a resistant attitude towards a green production activity and has not implemented it. The non-implemented refers to the peasant household that has not implemented a green production activity. The late implementer refers to the peasant household whose implementation of green production activity is at a later position in the village. The early implementer refers to the peasant household whose implementation of green production activity is at a higher position in the village. However, the earliest implementer refers to those who exercised the green production activity earliest among the farmers in the village. In the formal investigation, the investigators first asked farmers about the cognition and practice of three types of green production activities, such as (i) fertilizer and pesticide reduction, (ii) resource conservation such as straw returning to the field and biogas residue and slurry returning to the field, and (iii) environmentally friendly pest control such as the use of organic fertilizer and green prevention and control. Finally, green production demonstration households were categorized and characterized by pollution reduction, resource conservation, and environmentally friendly pest management demonstration households. Table 2 depicts the basic characteristics of the variables used in the study.

### 3.4. Basic Characteristics of Sample Farmers

Table 3 summarizes the basic characteristics of the sample farmers. In terms of green production demonstration households, 12.98% of the households were between those who resisted and those who did not implement it, 76.74% were between those who did not implement it and those who implemented it later, and 8.62% of farmers accounted for those farmers who are between late Implementer and earlier Implementer. Meanwhile 1.66% of farmers accounted for the earliest implementers. In terms of the embeddedness of government relations, 51.82% of the households accounted for between few and fewer interactions, 32.71% were less and average interactions, 13.50% were between normal and more interactions, and 1.97% of farmers accounted for significant interactions. In terms of the age of household heads, 12.77% of the household heads were 45 years old and below, 42.89% were 45–60 years old, and 44.34% were 60 years old and above. In terms of the internet proficiency of the household heads, 36.45% were very unfamiliar, 26.17% were less familiar, 26.06% were familiar, 9.14% were relatively familiar, and 2.18% were very familiar. In terms of business scale, 64.28% of households with 5 mu and below, 23.99% of farmers with 5 to 10 acres, and 11.73% of farmers with more than 10 acres. In terms of household technical status, 1.66% of households with very poor technical status, 13.60% of households with poor technical status, 67.08% of households with medium technical status, and 16.82% of households with good technical status. In terms of household economic status, 2.39 % of households with very poor economic status, 20.56% of households with poor economic status, 57.94% of households with medium economic status, 18.07% of households with good economic status, and 1.04% accounted for excellent. In terms of family political status, the proportion of party members’ families was only 11.94%.

### 3.5. Model Construction

#### 3.5.1. Benchmark Model

The green production demonstration households changed from low to high, which is an ordered variable, so the Ordered Probit model was used for estimation [127]. First, we defined a latent variable $Y^{*}$:

(1) $$Y^{*}=\alpha RS+\varepsilon$$

(2) $$Y=\left\{\begin{array}{l} w_{1}{,}_{}if\;Y^{*}\leq v_{1}\\ w_{2}{,}_{}if\;v_{1}<Y^{*}\leq v_{2}\\ \cdots \cdots \cdots \cdots \cdots \cdots \\ w_{i}{,}_{}if\;v_{i-1}<Y^{*}\leq v_{i} \end{array}\right.$$

In Equations (1) and (2), $Y^{*}$ is the latent variable, Y denotes green production demonstration households and is the observed variable, and RS represents the embeddedness of government relations and is the α coefficient of $v_{1},v_{2},\cdots v_{i-1},v_{i}$ which needed to be estimated and represented the cut point. The following model can be further obtained:

(3) $$P(Y=w_{1}|RS)=P(Y^{*}\leq v_{1}|RS)=P(v_{1}\leq \alpha RS+\varepsilon_{1}|RS)=\Phi {\left(v_{1}-\alpha RS\right)}_{}^{}$$

(4) $$P(Y=w_{2}|RS)=P(v_{1}<Y^{*}\leq v_{2}|RS)=\Phi (v_{2}-\alpha RS)-\Phi (v_{1}-\alpha RS)$$

(5) $$P(Y=w_{i}|RS)=P(Y^{*}>v_{i}|RS)=1-\Phi (v_{i}-\alpha RS)$$

#### 3.5.2. Mediating Effect Model

The government relationship embedding can act on the cultivation of green production demonstration households through intermediary variables such as farmers’ government identification, behavioral ability, and perceived value [56]. Therefore, the following equations were set to describe the relationship between the variables, for running the Bootstrap method, testing the mediation effect of the model, which can effectively solve the problem of the “masking effect” and reflect the real influence between variables [130]:

(6) $$Y=\alpha_{0}+\alpha_{1}RS+\tau_{1}$$

(7) $$M=\beta_{0}+\beta_{1}RS+\tau_{2}$$

(8) $$Y=\chi_{0}+\chi_{1}RS+\chi_{2}M+\tau_{3}$$

In Equations (6)–(8), Y is the green production demonstration household, RS is the government relationship embedding, and M is the intermediary variable.

## 4. Results

### 4.1. Benchmark Model Results and Analysis

STATA15.0 [StataCorp, https://www.stata.com (accessed on 27 June 2022)] regression and robust correction were used to rectify the benchmark of the estimated results. It can be seen from Table 4 that the overall fitting effect of the model was good and its explanatory power was strong [136]. The specific results and analysis are as follows. The results of Model 1, Model 2, and Model 3 show that the embedding of government relations has a positive impact on green production demonstration households at the 1% significance level. It also demonstrates that the government relationship embedding can enhance farmers’ trust in the government itself and foster better policy recognition, dispel farmers’ concerns about green production, and enable farmers to develop into green production demonstration households. Moreover, it enhances the farmers’ self-interest, facilitates the perception of social and altruistic value, enables the farmers to form an optimistic green production expectation that includes government actions, and leads them to develop green production demonstration households. In a study of the drivers of green agricultural development in the Chinese agrarian sector, Chen et al. [1] found similar findings.

The results of Model 4 show that the relationship embedding of general government staff, professional and technical personnel, and village cadres has a positive impact on the green production demonstration households at the 1% significance level, and the coefficient value gradually increases. The outcome is consistent with the study of Bukchin and Kerret [137]. Compared with general government staff, the relationship between village cadres is built based on generational familiarity and daily interaction and has a higher trust attribute. “Emotional mobilization” and “showing up” can make farmers recognize the practical value of the government and green production, and improve farmers’ awareness. Confidence in the successful realization of green production; compared with general government staff, professional and technical personnel have higher credibility due to their unshakable technical status. Therefore, the cultivating role of green production demonstration households embedded in the relationship between general government staff, professional and technical personnel, and village cadres is significant and increasing sequentially. These results are supported by the study of the policy impacts of green agriculture Wang et al. [11].

In Model 3 and Model 4, the proportion of non-agricultural income, age, and internet familiarity of the head of the household has a significant positive impact on the green production demonstration households. It shows that an older household head possesses relatively greater agricultural production experience and deeper affection for the land. Moreover, the regression analysis shows that their willingness and ability to implement green production are also high which eventually led them to become a green production demonstration household. This is parallel with the research of Li et al. [21]. Seemingly, internet information transmission is convenient and fast, and it is easier to obtain green production knowledge or opportunities, so the farmers familiar with the internet are more likely to become green production demonstration households. Similarly, farmers with a high degree of non-agriculturalization have more green production knowledge and more economic ability, so they are more likely to become green production demonstration households. This is supported by the findings of Mao et al. [30].

The status of household technology has a significant positive impact on the green production demonstration households. The farmers with a high level of tea planting technology are proficient in using green production literacy and skills, so they are more likely to become green production demonstration households. Interestingly, in their review study, Bukchin and Kerret [137] found similar outcomes and they highlighted the importance of interpersonal characteristics and technological attributes. In addition, household economic status has a significant negative impact on green production demonstration households. This may be because, with the increase in household income, the tendency of farmers to leave agriculture is highly coupled with the stable agricultural management strategy, and they are more cautious about green production, so they are reluctant to take the lead in implementing green production. Consumer quality sensitivity and participation in public affairs have a significant positive impact on green production demonstration households. The higher the consumer’s sensitivity to quality, the more pressure will be exerted on farmers’ production behavior, which can encourage farmers to develop into green production demonstration households. In a study of the tea industry of Assam, India, Deka et al. [43] depicted similar findings. While according to Soheili-Fard et al. [18], the early adoption of green production has positive externalities and a leading role in demonstration, so farmers with a higher enthusiasm for participating in public affairs are more likely to become green production demonstration households. Our study also found similar outcomes.

### 4.2. Robustness Test and Endogeneity Treatment

#### 4.2.1. Robustness Test

Based on the benchmark model, the robustness test is carried out in various ways. In Table 5, Model 5 adopts the Ordered Probit model with common standard error, in Model 6 and Model 7, respectively, we adopt the Ordered Logit model and multiple linear models to re-regress. We have re-regressed the outcomes in Model 8, Model 9, and Model 10 along with the dependent variable. The regression results of Model 5 to Model 10 show that the embedding of government relations has a positive impact on green production demonstration households at the 1% significance level, indicating that government relationship embedding has the effect of cultivating green production demonstration households. The outcome is consistent with the above regression results and indicates that the model results are robust [138].

#### 4.2.2. Endogenous Processing

The above models may suffer from endogeneity problems caused by simultaneous causal or omitted variables. This is because, first, there may be missing variables. Although the model controls variables such as household head characteristics, family characteristics, policy environment, and market environment, it may still miss some of the factors that are related to the embeddedness of government relations and affect green production demonstration households. Second, there may be a simultaneous bias problem. Government relationship embedding can cultivate farmers into green production model households through government identification, behavioral ability, and perceived value, and at the same time, the demonstration effect of green production model households can also counteract the government relationship embedding. The introduction of instrumental variables is an effective method to solve the problem of missing variables and simultaneous causality, as recommended by Mogstad and Wiswall [139]. The article selects “village road condition (very poor = 1, poor = 2, moderate = 3, good = 4, very good = 5)” as the instrumental variable embedded in government relations, which is obtained from village-level surveys. The structural representation of the village roads may usually depend on several factors, such as natural, historical, and strongly exogenous in nature. However, the villages with better road conditions are usually expected to be more convenient for communicating with the governmental services and have a higher degree of embeddedness in government relations. Since the latent endogenous variable government relationship embedding is a discrete variable, the IV O-Probit program cannot handle the situation, so the Extended Ordered Probit sub-block of Extended Regression Models are introduced for endogeneity processing, as suggested by Cameron and Trivedi [140] and the results are shown in Table 6. The results in Table 6 show that the Extended Ordered Probit model has a high overall significance and a good fitting effect as the *p* value of the correlation of the residuals between the equations is significant [141].

However, the government relationship embedding is indeed an endogenous variable. Therefore, such an endogeneity treatment method is reasonable. Interestingly, the village road condition (the only instrumental variable) has a significant positive effect on the embeddedness of government relations and has good explanatory power, which also proves that it is not a weak instrumental variable [139]. The results of the Probit model are consistent, indicating that the embeddedness of government relations has the effect of cultivating green production model households.

### 4.3. Analysis of the Mechanism of Action

To clarify the role of the mechanism of government relationship embedded in the cultivation of green production demonstration households, the 5000 sampling Bootstrap method is used to test the mediation effect, as recommended by Yang et al. [142]. The specific results are as follows:

(1) The coefficients of the two channel of government relationship embedded (“policy identification and green production demonstration households”, “government relationship embedded-government trust” and “green production demonstration households”) are positive, and they pass the 1% significance level test without containing 0 value. Therefore, it can be assumed that the government relationship embedding can play an important role in cultivating green production model households through policy recognition and government trust. The embedding of government relations is conducive to assisting farmers to facilitate green production behavior, alleviating conflicts and contradictions in the implementation of new policies, establishing a positive and promising image of the government, improving farmers’ recognition, and eventually building the farmer’s trust in the government and directly encouraging them to foster GPDH;

(2) The coefficients of the two paths (“government relationship embedding and self-efficient green production demonstration households”, and “government relationship embedding and production knowledge reserve green production demonstration households”) are positive, and they pass the 5% and 1% significance level tests, respectively. The value of 0 is not included, indicating that government relationship embedding can play a crucial role in cultivating green production demonstration households through self-efficacy and production knowledge reserves. Moreover, it also illustrates that the government relations embedding significantly enhances the communication between the government and farmers, and various policy measures such as persuasion, mobilization, education, and training can effectively enhance farmers’ production knowledge reserves and self-efficacy, and eventually encourage farmers to develop green production demonstration households;

(3) The coefficients of the three paths (“government relationship embedding, self-interested value perception, and green production demonstration households”, “government relationship embedding, altruistic value perception, and green production demonstration households”, and “government relationship embedding, social benefit perception, and green production demonstration households”) positively pass the 1% significance level test and the confidence interval not containing 0 value. It illustrates that the government relationship embedding influences the cultivation of green production demonstration households through the self-serving value perception, altruistic value perception, and social value perception. Table 7 represents the mediation of the effects of the core variables.

## 5. Conclusions

The diffusion and adoption of new and improved farming practices have provided a major area of research conducted by modern rural sociologists. Therefore, the notion of “innovation diffusion” becomes prominent within the agriculture sector. However, governmental interventions and support always account for a better transition of any innovation. The prime objective of the study is to test the influence and mechanism of government relationships embedded in fostering green production demonstration households by employing the expansion of the diffusion of innovation theory. Therefore, we constructed a theoretical framework that can firmly capture the direct and indirect influence of three forms of relational embeddedness, namely (i) Governmental organizations and bodies, (ii) Technical and professional support personnel, and (iii) Embeddedness of village cadres in fostering green production demonstration households. The empirical aspects of the article used the survey data of 963 farmers in key tea areas in Shaanxi, Sichuan, and Anhui for crafting the findings.

The main conclusions are as follows: (i) The embeddedness of government relations has a positive function in formulating green production demonstration households. In terms of dimensions, the green production demonstration households are embedded in the relationships of general government staff, professional and technical personnel, and village cadres, who all have a significant and increasing role in cultivating green production demonstration households. Therefore, we accepted H1 and H2. (ii) Heterogeneity analysis shows that the development and incentive effect of green production demonstration households embedded in government relations gradually increases with the expansion of the farmer’s business scale, and gradually weakens with the improvement of the economic status of the farmer’s household. The household head’s age, internet familiarity, and the proportion of non-agricultural income, family technical status, and family political status cannot effectively adjust the government relationship embedding in fostering green production demonstration households. Therefore, H3 was rejected. (iii) As the heterogeneity tests show that farmers with large operating scales and low family economic status have a relatively stronger impact, therefore the study partially verified H4 and H5. (iv) Further mechanism analysis showed that government relationship embedding can strengthen government identification (policy identification, government trust) and improve farmers’ behavioral ability (production knowledge reserve, self-efficacy). Moreover, formulating green production demonstration households can strengthen farmers’ perceived value (self-interest perception, altruistic value perception, and social value perception). Therefore H6–H8 were accepted.

Based on the above conclusions, the following specific policy recommendations can be drawn: (i) The government should strengthen the interactive mechanism embedded in the agricultural extension services. The embedding of government relations can help farmers correctly understand the government, policies, and green production itself, improve their abilities and qualities, and allow farmers to actively participate in green production practices and develop into green production demonstration households. Therefore, the government should strengthen relationship embedding by setting up new working methods such as “special work classes” and “assistance groups”, based on strengthening the interaction of village cadres, interactive and targeted rural policies, and consultation for opinions. The government should strengthen rural network facilities and build an interactive platform with the help of effective usage of information and communication technology to mitigate the limitations of time and space, and better play the embedded role of political and policy support, village cadres, and agricultural technicians;

(ii) The government should formulate targeted strategies based on the differential effect of green production demonstration households embedded in government relations under the scale of operation and household economic status. Likewise, the notion of government relationship embedding has greater impacts for those cultivating farmers with large operating scales and low family economic status. Therefore, in practice, the government should take these two types as the key training objects, continue to strengthen the relationship embedding between these two, and at the same time cooperate with large-scale management. Relevant policies, such as subsidies and technical support for farmers, should be transmitted through green production demonstration households, which can foster relatively better transitional effects;

(iii) Strengthen the mechanism of action embedded in government relations. In the relationship embedding, the government needs to grasp the two links of “knowledge” and “trust”. Information, such as policy and technical knowledge, needs to be effectively transmitted so that farmers can easily obtain the correct and legitimate information regarding governmental core objectives and policies. Therefore, they are more willing to actively implement green production and act as a new green production demonstration household. Government should organize purpose-based training through village cadres and extend support for on-the-spot training along with learning by watching and doing tactics. The effective circulation of bulletin boards, lectures, and demonstration videos should be extended to effectively help farmers in mastering scientific and technical skills. Seemingly, the targeted awareness-building campaigns, frequent arrangement of commendations, and mobilization meetings should be organized regularly to improve the farmer’s daily communication skills and enhance their self-efficacy as farmers and promote the development of the farmers’ knowledge about green production technology. The government should play the subjective role to promote the effect of green production through training, on-site demonstrations, and other means, so that farmers recognize the value of green products to allow the thriving of themselves, their communities, and society, and to encourage farmers to develop new green production demonstration households.

The study has the following main deficiencies and areas which can be explored by the potential studies: (i) China has a vast land mass and abundant resources, and there are differences in management methods and technical choices among farmers who grow different crops. The data used in the study are based on the central tea farming areas in Shaanxi, Sichuan, and Anhui. Therefore, the scope of the research objects should be expanded in the future to include farmers who produce different agricultural products in different regions; (ii) Variables such as green production demonstration households and government relationship embeddedness were obtained by a questionnaire survey. In future research, the crossover and integration with other disciplines should be strengthened, and the farmer household questionnaire survey should be combined with methods such as experiments, observations, and behavior traces; (iii) As the study compiled the key factors affecting the formulation of the green production demonstration household, future research should explore those factors with more robust tactics, such as structural equation modeling (SEM) and interpretive structural equation modeling; (iv) The study proposes that future studies should also explore the ranking and indexing of these critical factors into specific criteria.

## Figures and Tables

**Table 1:** Survey Area and Distribution of Sample Farmers.

Province	Shaanxi				Sichuan			Anhui		
County	Xixiang County	Ziyang County	Baihe County	Hanbin District	Wangcang County	Tongjiang County	Emeishan City	Jinzhai County	Qimen County	Huangshan District
Sample Size	81	107	111	81	91	100	103	97	94	98
Proportion (%)	8.41	11.11	11.53	8.41	9.45	10.38	10.70	10.07	9.76	10.18

**Table 2:** Variable Meaning and Basic Situation of Assignment.

Variable		Meaning and Assignment	Mean	SD
Dependent Variable				
Green Production Demonstration Households		Average Value of Three 5-Level Scales for Reduction Demonstration Households, Environment-Friendly Demonstration Households, and Resource Conservation Demonstration Households	2.6497	0.4963
Reduction Demonstration Household		Opponent = 1, Non-Implementer = 2, Late Implementer = 3, Early Implementer = 4, Earliest implementer = 5	2.3956	0.7453
Resource Conservation Demonstration Household		-	2.820 4	0.6301
Environmentally Friendly Demonstration Household		-	2.7331	0.8867
Independent Variable				
Government Relations Embedding		Average Value of Three 5-Level Scales for The Relationship Embedding of General Government Staff, the Relationship Embedding of Professional and Technical Personnel, and the Relationship Embedding of Village Cadres	2.2970	0.8048
General Staff Relationship Embedding		Few Interactions such as Information and Favors = 1, Few Interactions Such As Information and Favors = 2, Average Interactions such as Information and Favors = 3, More Interactions such as Information and Favors = 4, Many Interactions such as Information and Favors = 5	2.039 5	1.0127
Professional and Technical Personnel Relationship Embedded		-	2.2845	0.8271
Village Cadre Relationship Embedded		-	2.56 70	1.0878
Mediating Variable				
Government Approval	Government Trust	The Average Value of Three 5-Level Scales of Trust in County Government, Township Government, and Village Committee	3.4569	0.7496
	Policy Approval	Is the Green Production Policy in Line with Needs, Fairness, Acceptability, Execution, and Satisfaction with the Average of Five 5-Level Scales	3.1086	0.6862
Behavioral Capacity	Production Knowledge Reserve	Very Insufficient = 1, Less than Adequate = 2, Generally = 3, Somewhat Adequate = 4, Very Adequate = 5	3.0644	0.8699
	Self-Efficacy	Always Solve Difficult Problems, are More Interested in Difficult Problems, Overcome Frustration Quickly, Believe that there are Always More Solutions than Difficulties 4 Averages On A 5-Point Scale	3.4691	0.5693
Perceived Value	Self-Interested Value Perception	Green Production, Topics Increase, Response to Government Calls is Recognized, Green Production Leaves a Good Impression, and Personal Community Prestige Increases	3.0018	0.7806
	Altruistic Value Perception	Green Production Demonstration Helps Others: Very Small = 1, Small = 2, Average = 3, Large = 4, Very Large = 5	2.7975	1.0475
	Benefit Social Value Perception	The Average Value of Three 5-Level Scales of Green Production Necessity, Trend, and Benefit Based on Ecological Environment Protection and Product Quality Improvement	3.4039	0.6641
Control Variable				
Head of Household Characteristics	Age of Head of Household	Actual Age (Years)	57.3956	10.2728
	Homeowner’s Internet Familiarity	Very Unfamiliar = 1, Less Familiar = 2, Familiar = 3, Somewhat Familiar = 4, Very Familiar = 5	2.1443	1.0804
Family Business Characteristics	Business Scale	Actual Tea Garden Area (Mu)	6.1334	7.7548
	Share of Non-Agricultural Income	Nonfarm Income/Total Income	0.5370	0.3704
Family Status	Household Technical Status	Very Poor = 1, Poor = 2, Moderate = 3, Better = 4, Very Good = 5	3.0156	0.6357
	Family Economic Status	Very Poor = 1, Poor = 2, Moderate = 3, Better = 4, Very Good = 5	2.9481	0.7219
	Family Political Status	Number of Party Members in Agricultural Labor Force/Total Agricultural Labor Force	0.0632	0.1831
Policy Environment	Subsidy Policy	Government Subsidy/Total Household Income	0.0567	0.1145
	Normative Policy	Criticize the Policy, Prohibit the Policy, Check the Policy Implementation of the Average of 3 5-Level Scales	3.4185	0.6992
Market Environment	Consumer Quality Sensitivity	Very Insensitive = 1, Less Sensitive = 2, Average = 3, More Sensitive = 4, Very Sensitive = 5	3.0177	0.9815
Emotional Characteristics	Enthusiasm for Participation in Public Affairs	Very Bad = 1, Poor = 2, Fair = 3, Better = 4, Very Good = 5	3.0831	1.0873
	Place Attachment	Reputation for Hometown Tea: Very Little Care = 1, Less Care = 2, General Care = 5, More Care = 4, Very Care = 5	3.5358	0.8267
Sense of Gain	Life Satisfaction	The Average Value of the Three 5-Level Scales of High Family Life Quality, High Happiness, and Rushing in the Future	3.6068	0.5677

Note: Due to space limitations, the assignments of some variables are not shown in detail, and can be obtained from the author if necessary.

**Table 3:** Basic Characteristics of Sample Farmers.

Index	Options	Frequency	Proportion (%)	Index	Options	Frequency	Proportion (%)
Green Production Demonstration Households	(Green Production Resisters-not Implementers)	125	12.98	Government Relations Embedding	(Little Interaction—Less Interaction)	499	51.82
	(Non-Implementer—Late Implementer)	739	76.74		(Less Interaction—Average Interaction)	315	32.71
	(Late Implementer—Earlier Implementer)	83	8.62		(General Interaction—More Interaction)	130	13.50
	(Earliest Implementer)	16	1.66		(More Interaction—A Lot of Interaction)	19	1.97
Age of Head of Household	45 Years Old and Below	123	12.77	Household Technical Status	Very Poor	16	1.66
	45–60 Years Old	413	42.89		Poor	131	13.60
	60 Years Old and Above	427	44.34		Medium	646	67.08
Internet Proficiency of Households Head	Very Unfamiliar	351	36.45		Better	162	16.82
	Less Familiar	252	26.17		Very Good	8	0.83
	Familiar with	251	26.06	Family Economic Status	Very Poor	Twenty Three	2.39
	More Familiar	88	9.14		Poor	198	20.56
	Very Familiar	Twenty One	2.18		Medium	558	57.94
Business Scale	5 Acres and Below	619	64.28		Better	174	18.07
	5 Mu–10 Mu	231	23.99		Very Good	10	1.04
	More than 10 Acres	113	11.73	Family Political Status	Family without Party Members	848	88.06

**Table 4:** Results of Benchmark Model.

Variable		Model 1		Model 2		Model 3		Model 4	
		Green Production Demonstration Households		Green Production Demonstration Households		Green Production Demonstration Households		Green Production Demonstration Households	
		Coefficient	Rse	Coefficient	Rse	Coefficient	Rse	Coefficient	Rse
Government Relations Embedding		0.33 ***	0.0452	0.35 87 ***	0.0465	0.2840 ***	0.0515	-	-
General Staff Relationship Embedding		-	-	-	-	-	-	0.1325 ***	0.0435
Professional and Technical Personnel Relationships Embedded		-	-	-	-	-	-	0. 1503 ***	0.0515
Village Cadre Relationship Embedded		-	-	-	-	-	-	0.2023 ***	0.0407
Head Of Household Characteristics	Age of Head of Household	-	-	-	-	0.0106 **	0.0041	0.0106 **	0.0042
	Homeowner’s Internet Familiarity	-	-	-	-	0.1454 ***	0.0433	0.1450 ***	0.0437
Family Business Characteristics	Business Scale	-	-	-	-	−0.0435	0.0663	−0.0416	0.0667
	Share Of Non-Agricultural Income	-	-	-	-	0.3431 *	0.1860	0.3184 *	0.1862
Family Status	Household Technical Status	-	-	-	-	0.1800 ***	0.0640	0.1910 ***	0.0639
	Family Economic Status	-	-	-	-	−0.1232 **	0.0577	−0.1369 **	0.0580
	Family Political Status	-	-	-	-	0.2123	0.2584	0.1949	0.2593
Policy Environment	Incentives	-	-	-	-	0.1853	0.3829	0.1616	0.3858
	Normative Policy	-	-	-	-	0.0686	0.0516	0.0711	0.0521
Market Environment	Consumer Quality Sensitivity	-	-	-	-	0.0717 *	0.0385	0.0689 *	0.0388
Emotional Dimension	Participation in Public Affairs	-	-	-	-	0.1440 ***	0.0333	0.1500 ***	0.0339
	Place Attachment	-	-	-	-	0.0473	0.0475	0.0857	0.0744
Sense of Gain	Life Satisfaction	-	-	-	-	0.0780	0.0737	0.0507	0.0482
Regional Variable	Zihe, Hanbin, Wangcang, Tongjiang, Emeishan, Jinzhai, Qimen, Huangshan.	No		Yes		Yes		Yes	
Sample Size		963		963		963		963	
Waldchi2		55.17		140.79		213.85		228.73	
Prob		0.0000		0.0000		0.0000		0.0000	

Where: ***, **, and * represent the significance levels of 1%, 5%, and 10%, respectively.

**Table 5:** Robustness Test Results.

Variable	Model 5		Model 6		Model 7		Model 8		Model 9		Model 10	
	Ordered Probit Model		Ordered Logit Model		Multiple Linear Models		Ordered Probit Model		Ordered Probit Model		Ordered Probit Model	
	Green Production Demonstration Households		Green Production Demonstration Households		Green Production Demonstration Households		Reduction Demonstration Household		Environmentally Friendly Demonstration Household		Resource Conservation Demonstration Household	
	Coefficient	Standard Error	Coefficient	RSE	Coefficient	RSE	Coefficient	RSE	Coefficient	RSE	Coefficient	RSE
Government Relations Embedding	0.2840 ***	0.0495	0.5665 ***	0.0954	0.1223 ***	0.0226	0.2521 ***	0.0519	0.2207 ***	0.0547	0.2262 ***	0.0681
Control Variable	Yes		Yes		Yes		Yes		Yes		Yes	
Sample size	963		963		963		963		963		963	
Waldchi^2^/F value	212.05		220.36		8.42		253.93		148.79		132.47	
Probability	0		0		0		0		0		0	

Note: ***, **, and * represent the significance levels of 1%, 5%, and 10%, respectively.

**Table 6:** Endogenous Results.

Variable	Model 11		Model 1 2			
	Ordered Probit		Extended Ordered Probit			
	Green Production Demonstration Households		Government Relations Embedding		Green Production Demonstration Households	
	Coefficient	Standard Error	Coefficient	Standard Error	Coefficient	Standard Error
Government Relations Embedding	0.2840 ***	0.0515	-	-	1.2494 ***	0.0534
Hardened Roads in Villages	-	-	0.0413 *	0.0230	-	-
Corr (e. Government Relations Embedded, e. Green Production Demonstration Household)	-		0.0000			
Control Variable	YES		YES			
Sample Size	963		895			
Waldchi2	213.85		1721.58			
Prob	0.0000		0.0000			

Note: Due to the lack of some village-level data, the samples with missing values were deleted for Extended Ordered Probit. ***, **, and * represent the significance levels of 1%, 5%, and 10%, respectively.

**Table 7:** Results of Mediation Effect.

Path	Mediating Variable	Indirect Coefficient	Standard Error	Confidence Interval
Embedding Government Relations—Government Identification—Green Production Demonstration Households	Policy Approval	0.0069 ***	0.0016	[0.0037, 0.0101]
	Government Trust	0.0479 ***	0.0093	[0.0297, 0.0661]
Government Relationship Embedding—Behavioral Ability—Green Production Demonstration Household	Self-Efficacy	0.0393 ***	0.0097	[0.0204, 0.0583]
	Production Knowledge Reserve	0.0093 **	0.0038	[0.0018, 0.0168]
Government Relationship Embedding—Perceived Value—Green Production Demonstration Household	Self-Interested Value Perception	0.0518 ***	0.0134	[0.0255, 0.0780]
	Altruistic Value Perception	0.0120 ***	0.0037	[0.0048, 0.0193]
	Benefit Social Value Perception	0.0272 ***	0.0073	[0.0130, 0.0415]

Note: Due to the lack of some village-level data, the samples with missing values were deleted for Extended Ordered Probit. While ***, **, and * represent the significance levels of 1%, 5%, and 10%, respectively.

## Data Availability

The associated dataset of the study is available upon request to the corresponding author.
